# In Vivo Reflectance Confocal Microscopy of Basal Cell Carcinoma: Feasibility of Preoperative Mapping of Cancer Margins

**DOI:** 10.1111/j.1524-4725.2012.02587.x

**Published:** 2012-10-05

**Authors:** Zhan-Yan Pan, Jing-Ran Lin, Ting-Ting Cheng, Jia-Qiang Wu, Wen-Yu Wu

**Affiliations:** * Department of Dermatology, Huashan Hospital, Shanghai Medical College, Fudan UniversityShanghai, China; † Shanghai East Hospital, Tongji UniversityShanghai, China; ‡ Department of Dermatology, Jing'An District Centre Hospital of Shanghai (Huashan Hospital Fudan University Jing'An Branch)Shanghai, China

## Abstract

Reflectance confocal microscopy (RCM) images skin at cellular resolution and has shown utility for the diagnosis of nonmelanoma skin cancer in vivo. It has the potential to define lesion margins before surgical therapy.

**Objectives:**

To investigate the feasibility of RCM in defining the margins of basal cell carcinoma before surgery.

**Methods:**

The margins of 10 lesions were evaluated using RCM. Biopsies of the margins were used to confirm the results. A protocol was constructed to define margins. RCM was used to delineate preoperative surgical margins in 13 patients. Intraoperative frozen biopsy was used to confirm the margins.

**Results:**

In seven of 10 (70.0%) cases, the margins of the cancer were identified suing RCM. The tumor island was the critical feature in identifying the margins. In 12 of 13 (92.3%) cases, frozen biopsy corroborated that the surgical margins delineated by RCM were clear.

**Conclusion:**

RCM imaging of the margins is feasible and demonstrates the possibility of preoperative mapping of cancer margins.

The incidence of basal cell carcinoma (BCC) is steadily increasing in most countries, accounting for approximately 80% of all nonmelanoma skin cancers.^1^ Mohs micrographic surgery (MMS) has been shown to be the most efficacious technique for removal of aggressive high-risk BCC located on the mid-face and ears,^2^^,^^3^ but the technique is labor intensive and time consuming, so there is a need for effective tools for improving and facilitating accurate tumour demarcation in patients with these aggressive skin tumors.

In vivo reflectance confocal microscopy (RCM) enables the noninvasive imaging of superficial layers of the skin with high resolution that provides cellular detail.^4^ RCM has previously been reported to be useful in the in vivo evaluation of skin tumors such as BCC, squamous cell carcinoma,^5^ and malignant melanoma.^6^ The ability to use RCM to perform noninvasive evaluation of skin lesions means that it has the potential to define lesion margins before surgical therapy. It has been demonstrated recently that it is possible to examine nonmelanoma skin cancers in ex vivo tissue during Mohs micrographic surgery without frozen sections.^7^ The purpose of this study was to investigate the potential of RCM to define margins of BCC before surgery.

## Material and Methods

### Patients

Ten patients with lesions clinically suggestive of BCC and then biopsy proven were recruited randomly from the dermatology department for the margin study. Before the biopsy, lesional and adjacent nonlesional skin was examined using RCM. Thirteen patients with biopsy-proven BCC were recruited for surgical excision. The margins of these lesions needed to be flat enough for RCM examination. This study was conducted according to the Declaration of Helsinki Principles. Institutional approval and written informed consent were obtained.

### Instrumentation

Confocal imaging was performed using a commercially available near-infrared reflectance confocal laser scanning microscope (Vivascope 1500; Lucid Technologies, Henrietta, NY), which uses a diode laser with a wavelength of 830 nm and power of less than 15 mW. This system provides high-resolution images (horizontal resolution 1.0 μm, vertical optical section thickness 3.0 μm) from a depth of 0 to 250 μm in vivo (from the epidermis to the papillary dermis). A comprehensive description of this system has been reported previously.^8^

### RCM Imaging Protocol and Surgical Excision

A pilot study (the margin study) was conducted in the first 10 cases to assess qualitatively the best imaging conditions and to construct a protocol to define margins. Blocks of 2- by 2-mm mosaic image mode were used to detect the margins. Biopsies of the margins detected using RCM were performed for histopathologic analysis. Tumor islands were seen in most of the margins of the lesions,so we used this feature to delineate the margins.

**Figure 1:**
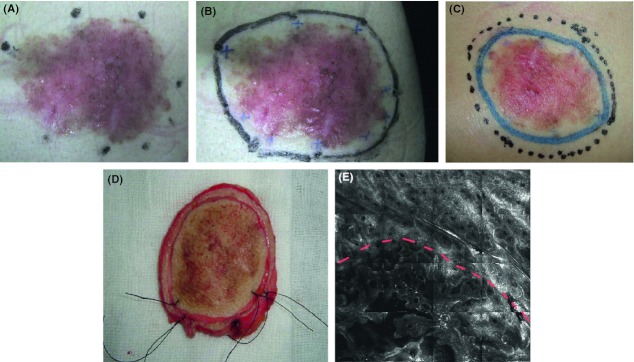
Reflectance confocal microscopy imaging protocol and surgical excision. (A) Foci for reflectance confocal microscopy (RCM) examination were selected. (B) The lesion's border was refined (blue cross). (C) An extra 5-mm margin of normal skin was excised (dotted line). (D) The gross skin specimen was further sectioned along the boundaries inscribed using RCM. (E) RCM mosaic image of the margin. The upper half part of the image showed the normal reticulated meshwork pattern, while the lower part showed the tumor islands of elongated strands.

The final protocol was as follows (Figure 1):

1. The lesion was evaluated by clinical inspection and palpation.
2. The lesion's border was delineated using a Wood's lamp and dermoscopy.
3. Selection of foci for RCM examination inside and outside the Wood's lamp and dermoscopy delineated border
4. Refinement of the lesion's border based on RCM findings. The feature of tumor islands was the key to identify the margin.
5. An extra 5-mm margin of normal skin was excised, and then intraoperative frozen biopsy was performed to ascertain the surgical margins.
6. The gross skin specimen was further sectioned along the boundaries inscribed by RCM and confirmed by intraoperative frozen biopsy.

### Histopathology

All biopsy specimens were routinely processed with formalin fixation and paraffin embedding followed by vertical sectioning and hematoxylin and eosin staining. Diagnoses were retrieved from the hospital information system. Slides were also examined for findings that appeared to correlate best with RCM structures under analysis.

## Results

### RCM Features of the Margin of the Lesions

**Figure 2:**
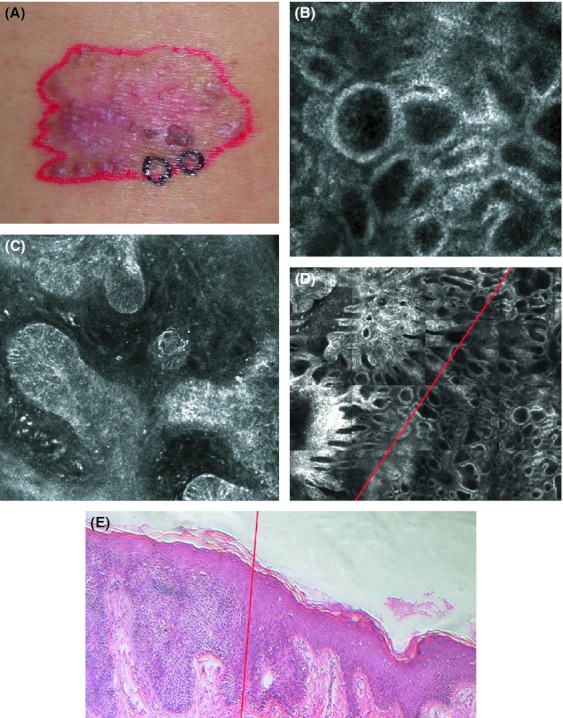
Reflectance confocal microscopy (RCM) images of the margin of a lesion. (A) Clinical image of a superficial basal cell carcinoma on the cheek of a 52-year-old woman. (B) RCM images of nonlesional skin showed the normal ring structures. (C) RCM images of lesional skin showed the tumor island. (D) RCM mosaic image of the margin. The left side of the image showed the tumor island, and the right side showed the normal ring structures. (E) Biopsies of the margins detected using RCM confirmed the result.

In seven of 10 (70%) cases, the margins of the cancer were identified. The RCM mosaics of the margins showed features of lesional and normal skin (Figure 2). Five RCM features of lesional skin were seen: atypical honeycomb at the level of epidermis, tumor islands at the dermal and basal layer, elongated monomorphic nuclei, increased vascularity, and prominent inflammatory cell infiltrate. Tumor islands, which can be observed most easily at the margin of the lesions, were seen in all cases. They are characterized by aggregates of atypical cells organized into nodules, islands, cords, or elongated strands. Because the normal side of the image was characterized by ring structures or reticulated meshwork at superficial dermis, the margin was distinct (Figure 2D). Histologic examination corroborated that the surgical margins identified using RCM were correct (Figure 2E). In three of 10 (30%) cases, the margin of the lesions could not be detected because of the unevenness of the surface.

### Surgical Excision

Thirteen patients were treated with local surgical excision. The data of the patients included are shown in Table 1. We observed nine (69.2%) BCC of the superficial type and four (30.8%) of the nodular type. Most BCC were located on the cheek. All frozen biopsies of surgical margins were negative. In 12 of 13 (92.3%) cases, frozen biopsy of RCM margins was negative. The RCM margin of a nodular type BCC case was positive. Cancer cells were found under the surface of one section.

**Table 1:** Clinical Data of 13 Basal Cell Carcinomas (BCC) and Histopathologic Results of Surgery

Patient	BCC Type	Location	Clinical Size, cm	Frozen Biopsy of Surgical Margins	Frozen Biopsy of Reflectance Confocal Microscopy Margins
1	Superficial	Back	3.0 × 2.5	Negative	Negative
2	Superficial	Cheek	2.5 × 2.5	Negative	Negative
3	Nodular	Nose	0.8 × 0.8	Negative	Negative
4	Superficial	Back	1.5 × 2.5	Negative	Negative
5	Superficial	Cheek	0.9 × 0.9	Negative	Negative
6	Superficial	Cheek	1.0 × 1.0	Negative	Negative
7	Superficial	Leg	6.1 × 5.0	Negative	Negative
8	Superficial	Forehead	2.0 × 1.8	Negative	Negative
9	Nodular	Cheek	0.8 × 0.8	Negative	Positive
10	Nodular	Cheek	1.0 × 0.5	Negative	Negative
11	Nodular	Ear	0.8 × 0.5	Negative	Negative
12	Superficial	Cheek	0.5 × 0.7	Negative	Negative
13	Superficial	Nose	0.8 × 0.7	Negative	Negative

## Discussion

RCM has proven to be a promising, noninvasive, high-resolution imaging tool for histologic evaluation of the skin in vivo. The major microscopic features of several premalignant and malignant skin lesions have recently been reported.^9^^–^^12^ A few studies have confirmed that RCM offers a sensitive and specific tool for the noninvasive diagnosis of BCC in vivo.^13^^,^^14^

One potential application of RCM is to assess cancer margins. RCM may aid in the rapid establishment of tumor margins by examining excised specimens during procedures such as Mohs micrographic surgery.^7^ With RCM of ex vivo unprocessed tissues, it is possible to detect neoplastic cells using 5% acetic acid.^15^ It has also been demonstrated recently that RCM can define lesion margins, such as amelanotic melanomas^6^ and extramammary Paget disease,^16^ before surgery.

We showed the feasibility of using RCM to identify the tumor margins and corroborated this finding through histologic examination of the surgical margins identified using RCM. RCM features of BCC have been previously described.^17^ With RCM, BCCs exhibit five confocal features, regardless of subtype. Our study showed that the tumor island was the critical feature in identifying the margins. The RCM mosaics of the margins showed features of lesional and normal skin. The margin of the lesions was distinct in the picture. We constructed a protocol to define the margins according to the margin study and the literature.^18^ We have presented 13 cases in which RCM was used to guide surgical excision of BCC. The current study showed that this technique can be applied in superficial and nodular types of BCC. Frozen biopsy confirmed the accuracy of the margins delineated using RCM.

The present study has several limitations. The main limiting factor has been the limited depth of RCM penetration, which prevents accurate imaging at depths below the superficial dermis. The limited depth of RCM penetration accounted for the positive case of nodular type BCC. Second, this technique requires that the surface of the margin be flat enough for examination. Hyperkeratosis, the thickness, and the anatomic position of lesions may make examination difficult. Morphea-type BCC was not studied.

In summary, we have demonstrated that RCM can help to delineate surgical borders in a common skin cancer: BCC. In cases involving a large area of lesional skin, margins mapped out using RCM should help Mohs micrographic surgeons in identifying surgical margins. Future studies involving larger sample sizes and histologically different BCC subtypes will be needed to further elucidate the full utility of this technique.

## References

[b1] English DR, Armstrong BK, Kricker A, Fleming C (1997). Sunlight, cancer. Cancer Causes Control.

[b2] Scope A, Mahmood U, Gareau DS, Kenkre M (2010). In vivo reflectance confocal microscopy of shave biopsy wounds: feasibility of intraoperative mapping of cancer margins. Br J Dermatol.

[b3] Jih MH, Friedman PM, Goldberg LH, Kimyai-Asadi A (2005). Curettage prior to Mohs' micrographic surgery for previously biopsied nonmelanoma skin cancers: what are we curetting? Retrospective, prospective, comparative study. Dermatol Surg.

[b4] Calzavara-Pinton P, Longo C, Venturini M, Sala R (2008). Reflectance confocal microscopy for in vivo skin imaging. Photochem Photobiol.

[b5] Rishpon A, Kim N, Scope A, Oliviero MC (2009). Reflectance confocal microscopy criteria for squamous cell carcinomas, actinic keratoses. Arch Dermatol.

[b6] Curiel-Lewandrowski C, Williams C, Swindells K, Tahan SR (2004). Use of in vivo confocal microscopy in malignant melanoma: an aid in diagnosis, assessment of surgical, nonsurgical therapeutic approaches. Arch Dermatol.

[b7] Patel Y, Nehal K, Aranda I, Li Y (2007). Confocal reflectance mosaicing of basal cell carcinomas in Mohs surgical skin excisions. J Biomed Opt.

[b8] Gonzalez S, Gilaberte-Calzada Y (2008). In vivo reflectance-mode confocal microscopy in clinical dermatology, cosmetology. Int J Cosmet Sci.

[b9] Aghassi D, Anderson RR, Gonzalez S (2000). Confocal laser microscopic imaging of actinic keratoses in vivo: a preliminary report. J Am Acad Dermatol.

[b10] Gerger A, Koller S, Weger W, Richtig E (2006). Sensitivity, specificity of confocal laser-scanning microscopy for in vivo diagnosis of malignant skin tumors. Cancer.

[b11] Braga JC, Scope A, Klaz I, Mecca P (2009). The significance of reflectance confocal microscopy in the assessment of solitary pink skin lesions. J Am Acad Dermatol.

[b12] Ulrich M, Maltusch A, Rius-Diaz F, Rowert-Huber J (2008). Clinical applicability of in vivo reflectance confocal microscopy for the diagnosis of actinic keratoses. Dermatol Surg.

[b13] Nori S, Rius-Díaz J F, Cuevas J, Goldgeier M (2004). Sensitivity, specificity of reflectance-mode confocal microscopy for in vivo diagnosis of basal cell carcinoma: a multicenter study. J Am Acad Dermatol.

[b14] Marra D, Torres A, Schanbacher C, Gonzalez S (2005). Detection of residual basal cell carcinoma by in vivo confocal microscopy. Dermatol Surg.

[b15] Rajadhyaksha M, Menaker G, Flotte T, Dwyer P (2001). Confocal examination of nonmelanoma cancers in thick skin excisions to potentially guide mohs micrographic surgery without frozen histopathology. J Invest Dermatol.

[b16] Pan ZY, Liang J, Zhang QA, Lin JR (2012). In vivo reflectance confocal microscopy of extramammary Paget disease: diagnostic evaluation and surgical management. J Am Acad Dermatol.

[b17] Sauermann K, Gambichler T, Wilmert M, Gonzalez S (2002). Investigation of basal cell carcinoma [correction of carcionoma] by confocal laser scanning microscopy in vivo. Skin Technol Res.

[b18] Ardigo M, Ardigo M, Delgado R, Gonzalez S (2008). Reflectance confocal microscopy of cutaneous tumors:an atlas with clinical,dermoscopic, histological correlations.

